# Concussion risk and symptomology severity in adolescents are associated with pre-season drowsiness and emotional complaints

**DOI:** 10.17159/2078-516X/2024/v36i1a16255

**Published:** 2024-07-15

**Authors:** D Stevens, C Grant, T Botha, G Vosloo, H Rossouw, P De Jager, L Holtzhausen

**Affiliations:** 1 Flinders Health and Medical Research Institute, Flinders University, South Australia, Australia; 2 Section Sports Medicine, Faculty of Health Sciences, University of Pretoria, Pretoria, South Africa; 3 Department of Statistics, Faculty of Natural and Agricultural Sciences, University of Pretoria, Pretoria, South Africa; 4 Sport Medical Service, Loftus, Pretoria, South Africa; 5 Sports Medicine Department, Aspetar Orthopaedic and Sports Medicine Hospital, Doha, Qatar; 6 Department of Exercise and Sports Science, University of the Free State, Bloemfontein, South Africa

**Keywords:** mild traumatic brain injury, sleep, mental health

## Abstract

**Background:**

Concussions are an ever present risk for many sports. Underlying emotional disturbances and drowsiness are associated with worse post-concussion symptom scores. Yet, not study has examined associations of both emotional disturbances and drowsiness on concussion severity and symptomology.

**Objectives:**

Examine the associations between baseline sleepiness, emotional complaints, and concussion risk and symptom severity in adolescent athletes.

**Methods:**

A cohort of 626 adolescent athletes underwent baseline/pre-season concussion screening. Those who experienced a physician diagnosed concussion underwent follow up concussion symptomology assessment. Over 90% of players were seen within two weeks of the concussion incident. Linear regression examined for associations between concussion symptom scores and baseline drowsiness and baseline emotional symptoms. Logistic regression examined for association between each symptom and baseline drowsiness and baseline emotional symptoms.

**Results:**

Of the 626 athletes that underwent baseline testing, 292 experienced a concussion. Of those 292 athletes, 174 (59.6%) reported baseline drowsiness and 183 (62.7%) baseline emotional symptoms. Baseline drowsiness and emotional complaints were associated with a 2.6 (95% confidence interval = 1.9 to 3.6) and 2.8 (95% confidence interval = 2.0 to 3.9) times greater odds of sustaining a concussion respectively. Increased symptomology after concussion was associated with both baseline drowsiness (unstandardised b = 4.6, p < 0.01) and baseline emotional complaints (unstandardised b = 6.0, p < 0.01).

**Conclusion:**

Preseason drowsiness and emotional complaints in adolescent athletes are associated with higher risk of adverse clinical outcomes following concussion. Therefore, clinicians and coaches should be aware, and properly screen, for sleep and emotional problems as part of pre-season/baseline health screening.

A concussion occurs when the brain experiences a sudden linear or rotational acceleration/deceleration of the head relative to the body. This acceleration-deceleration causes a shearing axonal injury which triggers a neuro-metabolic cascade. Unsurprisingly, athletes in collision sports such as rugby union are at a high risk of sustaining a concussion.^[1]^ Repeated concussions may present a long-term risk to neurological health, which is why concussions experienced during adolescence (typically 12 to 18 years old) are particularly concerning. In 2016, close to 20% of a cohort of adolescents reported experiencing at least one concussion in their life, whilst 5.5% reported experiencing multiple concussions.^[2]^ Furthermore, there have been year-on-year increase in the number of concussions reported by adolescent athletes.^[3]^

The identification and subsequent diagnosis of concussion relies heavily on symptom questionnaires, which examine areas of emotions, clarity of thought, sleep, fatigue, and vision problems. Yet these areas are also susceptible to underlying emotional disturbances, such as depression and anxiety, and drowsiness, which is largely due to sleep disturbances. Importantly, emotional disturbances and drowsiness are prevalent in adolescents.^[4]^

Within adolescents, pre-concussion sleep problems are associated with worse post-concussion symptom scores, neurocognitive impairment, with recovery from symptoms and neurological impairment taking longer than those without pre-concussion sleep problems.^[5]^ Likewise, pre-concussion emotional disturbances, including depression and anxiety, were also associated with worse post-concussion symptom scores, and again longer recovery from these symptoms compared to those without pre-concussion emotional disturbances.^[6]^ Yet to our knowledge, no study has examined both sleep and emotional disturbances simultaneously. Therefore, this study sought to elucidate associations between baseline sleep and emotional symptoms with concussion severity and symptomology in adolescent athletes (10–18 years old).

## Methods

### Participants

Baseline data from 626 adolescent participants (age = 15.5 ± 1.7 years) were prospectively collected from a single sports medicine clinic (Pretoria, RSA) from 2016–2020. The University of Pretoria Human Research Ethics Committee approved analysis of the deidentified data (protocol number 486/2020).

### Baseline and post-concussion symptomology assessment

All participants underwent concussion symptomology assessment (COGNIGRAM®, Cogstate, Vic., Australia) as part of standard baseline/pre-season assessment. Participants who were diagnosed with concussion by a physician at the sports medicine clinic underwent the follow up concussion symptomology assessment testing at the same time. The concussion symptomology assessment identifies and grades 24 symptoms (each listed in Table 2) from zero (no symptoms) to six (very severe).

### Baseline sleep and emotional disturbance assessment

Participants were judged to be experiencing drowsiness at baseline assessment if they responded to having any “*drowsiness*” symptoms (score of 1 or more). Likewise, participants were judged to have baseline emotional symptoms by averaging the responses to both “*more* *emotional*” and “*irritability*” symptoms (average score of 1 or more).

### Statistical analysis

All data analysis was completed in R (R Foundation for Statistical Computing, v4.0.3, Vienna, Austria). Linear regressions determined associations between overall concussion symptom scores and 1) baseline drowsiness, 2) baseline emotional symptoms, and 3) the combination of both baseline drowsiness and emotional symptoms. Logistic regressions determined associations between all concussion symptoms and baseline drowsiness (Model 1), baseline emotional symptoms (Model 2) and the combination of both baseline drowsiness and emotional symptoms (Model 3). All models presented unstandardised β (B), which shows the relationship between the predictor variable and the dependent variable after adjustments, significance which was set at p < 0.05, and 95% confidence interval (CI). All linear regressions and logistic regressions adjusted for; age, time from injury, number of previous concussions, loss of memory during any previous concussion, and loss of consciousness during any previous concussion. These adjustments were selected given their known influence on concussion symptomology.^[7–9]^

## Results

### Participants

Of the 626 participants recorded at baseline, 292 (15.7 ± 1.69 years, 281 [96.2%] males) sustained a concussion and therefore completed a post-injury concussion symptomology assessment, with 265 participants (91%) completing the follow-up testing within two weeks of the injury. For those who experienced multiple concussions after baseline testing, the most recent concussion was used in the analysis. Of the 292 diagnosed with a concussion, 174 (59.6%) reported baseline drowsiness, whilst 183 (62.7%) reported baseline emotional symptoms.

### Associations and odds ratios of sustaining a concussion for baseline drowsiness and baseline emotional symptoms

The presence of baseline drowsiness was associated with increased concussion symptom scores (B = 4.6, p = 0.01, 95% CI = 1.2 to 7.9) and was associated with a 2.6 times greater odds of sustaining a concussion, compared to those who did not report baseline drowsiness (95% CI = 1.9 to 3.6) (Table 1). The presence of baseline emotional symptoms was associated with increased total concussion symptom scores (B = 6.0, p < 0.001, 95% CI = 2.7 to 9.4), and was associated with a 2.8 times greater odds of sustaining a concussion (95% CI = 2.0 to 3.9) compared to those who did not report baseline emotional symptoms (Table 1).

When both baseline drowsiness and baseline emotional symptoms were adjusted within the same model (Table 1), the presence of baseline emotional symptoms was associated with increased concussion symptom scores (B = 5.0, p = 0.006, 95% CI = 1.5 to 8.6), whereas the presence of baseline drowsiness was not (B = 2.8, p = 0.12, 95% CI = −0.7 to 6.3).

### Associations of baseline drowsiness and emotional symptoms on post-concussion symptomology

Associations between all concussion symptoms and post-concussion drowsiness, emotional symptoms, and a combination of the latter two are presented in Table 2. Respectively, baseline drowsiness was associated with 13/24 symptoms (Model 1), whilst baseline emotional symptoms were associated with 17/24 symptoms (Model 2). When examining both baseline drowsiness and emotional symptoms examined together in the linear regression (Model 3), baseline drowsiness was associated with seven symptoms, whilst baseline emotional symptoms were associated with 11 symptoms. Interestingly, only four symptoms, being ‘Balance problems or dizzy, ‘drowsiness’, ‘more emotional’, and ‘difficulty concentrating’, were simultaneously associated with both baseline drowsiness and emotional symptoms.

## Discussion

This study indicates that in a cohort of adolescent athletes, the presence of both baseline drowsiness and emotional symptoms are associated with an increased risk of concussion and higher concussion symptom scores. Likewise, worsening baseline sleep and emotional symptoms were associated with increased reporting of post-concussive symptoms.

The findings that drowsiness and emotional complaints individually are associated with concussion risk and symptoms supports previous studies.^[5,6]^ Importantly, this study was the first to examine both drowsiness and emotional complaints together within regression models that adjusted for other known confounders of concussion, such as previous concussion history.

The finding of the baseline drowsiness analysis demonstrating associations with both a greater risk of sustaining a concussion, and a range of post-concussion symptoms, confirms findings of Surfinko *et al*.,^[5]^ despite differences in population and statistical analysis used. This study also expands on recent findings of Raikes *et al*., which showed players who reported insomnia symptoms had greater odds of sustaining a concussion.^[10]^

Similarly, the finding that baseline emotional complaints is associated with both a greater risk of sustaining a concussion, and a range of post-concussion symptoms, confirms findings of Green et al.,^[6]^ again despite differences in population and statistical analysis used. Somewhat surprisingly, however, there appears to be no research examining the effect of baseline emotional disturbances, including mental health problems, on concussion risk and severity in adult populations, despite mental health concerns impacting baseline screening of concussion.^[11]^

An interesting finding was the outcome of the model adjusting for both baseline sleep complaints and emotional disturbances, where only baseline emotional disturbances was associated with greater odds of concussion. Given sleep disturbances mediate emotional disturbances,^[12]^ this could explain the lack of association between baseline drowsiness complaints and concussion risk when also adjusting for baseline emotional complaints.

Due to the lack of objective concussion-specific assessment tools, neurological mechanisms to explain the results cannot be determined. Nevertheless, there are several possible mechanisms that could explain the results shown. Neurologically, underlying dysfunction of the limbic system may increase the odds of experiencing a concussion, and worse symptom reporting. The limbic system regulates both sleep and emotions, primarily through the amygdala,^[13]^ and sleep via the suprachiasmatic nucleus located in the hypothalamus. 14 Traumatic brain injuries result in chronic changes to neural circuitry within the limbic system,^[15,16]^ resulting in ongoing emotional disturbances.^[17]^ Retired National Football League players demonstrated reduced limbic system volumes compared to age matched controls who have not participated in collisions sports,^[18]^ but it is unclear whether these reductions were present prior to, or as a result of, concussion. To address this, future studies should undertake baseline and post-concussion neuroimaging to examine potential neurological correlates of concussion. Neuropsychologically, sleep and emotional disturbances have been independently associated with increased musculoskeletal injuries.^[19]^ It is hypothesised both sleep and emotional disturbances may impair vigilance and impair skill performance, meaning that players put themselves in positions which increase the likelihood of injury. It is reasonable to assume that the same processes could also increase the likelihood of sustaining a concussion. Further research is needed to further elucidate this hypothesis.

Also of note, and not part of the original aims of the study, was the high level of concussion shown in this population. The authors suspect that a combination of good knowledge of concussion risk, and vigilance by those involved within sports where this population were involved, meant that all players with a suspected concussion were referred for clinical follow up. Sport is currently plagued with ‘missed concussions’, so the increased awareness seemingly demonstrated in this study may potentially lead to a reduction in these missed concussions.^[20]^

The strengths of this study were the large patient cohort utilising the same validated concussion assessment tools for all participants at baseline and follow up. A further strength is the follow-up concussion symptomology assessments occurring relatively soon after injury.

This study did have weaknesses that can be used to guide future research. The first weakness is the lack of objective sleep measures, such as actigraphy and overnight polysomnography, as well as the lack of validated mental and emotional health questionnaires. Thus, future studies, as well as clinical practice, should implement these measures. This will allow for in depth examination of whether specific sleep factors, such as a type of sleep disorder, or emotional factors, such as emotional intelligence and self-awareness, influence symptom reporting and concussion severity. This information will also allow clinicians to intervene if any clinical issue presents, potentially reducing the risk and severity of future concussions. A further shortcoming is not being able to adjust for other variables that may affect both drowsiness and emotional complaints, including medications such as antidepressant and sedatives. These weaknesses can be addressed by implementing objective sleep measures, as well as validated mental health questionnaires, into future research and clinical practice. We acknowledge the majority of concussions in this study occurred in males, making between-gender analysis difficult. There has been rapid growth of female participation in collision sports in recent times, meaning future studies should have larger proportion of females from which between-gender analyses can occur.

Future research should also examine whether baseline drowsiness and emotional complaints affects recovery times. This will help determine whether those with baseline drowsiness and emotional complaints require different interventions after concussion.

## Conclusion

This study provides novel evidence that sleep problems and emotional disturbances can precipitate, and exacerbate, concussion. Therefore, proper screening of sleep and emotional symptoms in adolescent athletes should become standard practice to help identify those who may be at risk of sustaining a concussion, or who may experience worse symptoms of concussion.

## Figures and Tables

**Table 1:** The relationship between baseline drowsiness and emotional complaints with the odds of sustaining a concussion

		Coefficient (95% CI)	p-value	Odds of sustaining a concussion (95% CI)
**Baseline drowsiness**		4.6 (1.2–7.9)	0.0081*	2.6 (1.9 – 3.6)
**Baseline emotional complaints**		6.0 (2.7 – 9.4)	0.0005*	2.8 (2.0 – 3.9)
**Baseline drowsiness and baseline emotional complaints**	**Drowsiness**	2.8 (−0.7 – 6.3)	0.1200	
	**Emotional complaints**	5.0 (1.5 – 8.6)	0.0060*	

* indicates significant association with symptom.

All models adjusted for age, time from injury, number of previous concussions, loss of memory during most recent concussion, and loss of consciousness during most recent concussion. CI, confidence interval.

**Table 2:** Logistic regression associations between concussion symptoms and baseline drowsiness (Model 1), baseline emotional symptoms (Model 2) and the combination of both baseline drowsiness and emotional symptoms (Model 3)

Clinical symptom	Model 1	Model 2	Model 3	

			Baseline drowsiness	Baseline emotional complaints

Headache	0.2668	0.3054	0.4212	0.4961
Pressure in head	0.2793	0.1103	0.5750	0.1927
Neck pain	0.5662	0.0410*	0.8655	0.0489*
Balance problems or dizzy	0.0043*	0.0036*	0.0459*	0.0383*
Nausea or vomiting	0.2548	0.1059	0.5435	0.1904
Vision problems	0.2429	0.0320*	0.6583	0.0627
Hearing problems/ringing	0.0308*	0.0088*	0.1801	0.0426*
Don’t feel right	0.4549	0.1428	0.8029	0.1982
Dinged/dazed	0.0148*	0.0446*	0.0624	0.2157
Confusion	0.0327*	0.0032*	0.2408	0.0175*
Feeling slowed down	0.0595	0.0336*	0.2228	0.1166
In a fog	0.0222*	0.0205*	0.1109	0.0995
Drowsiness	0.0008*	0.0023*	0.0133*	0.0395*
Fatigue or low energy	0.1336	0.4929	0.1786	0.8631
More emotional	0.0006*	<0.0001*	0.0204*	0.0022*
Irritability	0.0022*	<0.0001*	0.2719	<0.0001*
Difficulty concentrating	0.0020*	0.0025*	0.0271*	0.0357*
Difficulty remembering	0.0718	0.0443*	0.2436	0.1398
Sadness	0.1518	0.0014*	0.7614	0.0032*
Nervous or Anxious	0.0274*	0.0002*	0.3296	0.0013*
Trouble falling asleep	0.0135*	0.0733	0.0474*	0.3155
Sleeping more than usual	0.0008*	0.1762	0.0020*	0.8516
Sensitivity to light	0.1226	0.0130*	0.4696	0.0369*
Sensitivity to noise	0.0034*	0.0167*	0.0242*	0.1380

Data are expressed as p-values.

* indicates significant association with symptom.

All models adjusted for age, time from injury, number of previous concussions, loss of memory during most recent concussion, and loss of consciousness during most recent concussion.

## Data Availability

Due to the age of participants, data are not publicly available, however, deidentified data can be provided upon reasonable request to the corresponding author.

## References

[1] Daneshvar DH, Nowinski CJ, McKee AC, Cantu RC (2011). The epidemiology of sport-related concussion. Review Clin Sports Med.

[2] Veliz P, McCabe SE, Eckner JT, Schulenberg JE (2017). Prevalence of concussion among us adolescents and correlated factors. JAMA.

[3] Zhang AL, Sing DC, Rugg CM, Feeley BT, Senter C (2016). The rise of concussions in the adolescent population. Orthop J Sports Med.

[4] Polanczyk GV, Salum GA, Sugaya LS, Caye A, Rohde LA (2015). Annual research review: A meta-analysis of the worldwide prevalence of mental disorders in children and adolescents. J Child Psychol Psychiatry.

[5] Sufrinko A, Pearce K, Elbin RJ (2015). The effect of preinjury sleep difficulties on neurocognitive impairment and symptoms after sport-related concussion. Am J Sports Med.

[6] Green KE, Purtzki J, Chapman A, Oberlander TF, Silverberg ND, Dhariwal AK (2022). Somatization in adolescents with persistent symptoms after concussion: A retrospective chart review. J Neuropsychiatry Clin Neurosci.

[7] Roy D, Peters ME, Everett A (2019). Loss of consciousness and altered mental state predicting depressive and post-concussive symptoms after mild traumatic brain injury. Brain Inj.

[8] McCrea M, Guskiewicz K, Randolph C (2013). Incidence, clinical course, and predictors of prolonged recovery time following sport-related concussion in high school and college athletes. J Int Neuropsychol Soc.

[9] Corwin DJ, Zonfrillo MR, Master CL (2014). Characteristics of prolonged concussion recovery in a pediatric subspecialty referral population. J Pediatr.

[10] Raikes AC, Athey A, Alfonso-Miller P, Killgore WDS, Grandner MA (2019). Insomnia and daytime sleepiness: Risk factors for sports-related concussion. Sleep Med.

[11] Weber ML, Dean JL, Hoffman NL (2018). Influences of mental illness, current psychological state, and concussion history on baseline concussion assessment performance. Am J Sports Med.

[12] Barclay NL, Gregory AM (2013). Quantitative genetic research on sleep: a review of normal sleep, sleep disturbances and associated emotional, behavioural, and health-related difficulties. Sleep Med Rev.

[13] Heimer L, Van Hoesen GW (2006). The limbic lobe and its output channels: implications for emotional functions and adaptive behavior. Neurosci Biobehav Rev.

[14] Mistlberger RE (2005). Circadian regulation of sleep in mammals: role of the suprachiasmatic nucleus. Brain Res Rev.

[15] Ewing-Cobbs L, Johnson CP, Juranek J (2016). Longitudinal diffusion tensor imaging after pediatric traumatic brain injury: Impact of age at injury and time since injury on pathway integrity. Hum Brain Mapp.

[16] Keightley ML, Sinopoli KJ, Davis KD (2014). Is there evidence for neurodegenerative change following traumatic brain injury in children and youth? A scoping review. Front Hum Neurosci.

[17] Ewing-Cobbs L, DeMaster D, Watson CG (2019). Post-traumatic stress symptoms after pediatric injury: relation to pre-frontal limbic circuitry. J Neurotrauma.

[18] Lepage C, Muehlmann M, Tripodis Y (2019). Limbic system structure volumes and associated neurocognitive functioning in former NFL players. Brain Imaging Behav.

[19] Huang K, Ihm J (2021). Sleep and injury risk. Curr Sports Med Rep.

[20] Kroshus E, Garnett B, Hawrilenko M, Baugh CM, Calzo JP (2015). Concussion under-reporting and pressure from coaches, teammates, fans, and parents. Soc Sci Med.

